# On the Antiquity of Syphilis

**Published:** 1818-09

**Authors:** 


*For the London Medical and Physical Journal.*

### *On the Antiquity of Syphilis;* by Mr. Beetson.

AN inaugural Thesis, written many years since by Dr. Burder, on Syphiloid Diseases, has been translated by a cotemporary Journal, affording, in the opinion of the editors, some light upon an important subject of discussion which now occupies the attention of the profession.

This Essay appears to me to be entitled to the most attentive consideration, as the production of an inquisitive and accurate observer, and, probably, as a record of the practice and opinions of the surgeons of the Lock Hospital; but it seems to have been brought forward for the purpose of supporting the opinions of the mercurialists,—that the cases of syphilis, said to have been cured without mercury, are not genuine examples of that disease, but are of the species generally known by the term Syphiloid, or Pseudo-syphilis. Here then we are at issue, and this is the great point on which the question must turn.

It will be observed, on an examination of these cases, published in your last Number, that a great proportion of them arose in consequence of connexion with women, either known or suspected to be infected.—Are there then two species of ulcerative disease of the genitals consequent to venereal contact so accurately resembling each other as not to be distinguishable by mere observation, one of which is the genuine syphilis curable only by mercury, the other a poison of a different kind, which either wears itself out or yields to the influence of sarsaparilla and medicines of like properties? Or, is it the same disease, varied only as existing in different habits, or modified by peculiar circumstances? The author is inclined to believe syphiloid disease a complaint quite distinct from syphilis, sometimes arising spontaneously and without apparent cause, sometimes excited by various diseased secretions of the genitals, and from the matter of virulent gonorrhœa. It existed, he observes, "*long before syphilis was known:*" the latter not having been described more than about three centuries, though diseases of the genitals have been observed *ab initio mundi.*

The author then takes it for granted, with some others, that venereal disease was not known until within about three centuries: the year 1494 is fixed upon by Astruc as the period when the new light dawned upon mankind, when the *nova pestis* appeared; although complaints of the genitals had existed as far back as the earliest records of medicine. As the proper consideration of this subject bears strongly on the question respecting the power of curing syphilis without mercury, I subjoin some quotations from the older authors, which indisputably prove its existence anterior to the period generally assigned for its introduction; and, it will be observed, that if this be true, and the disease was actually cured before the efficacy of mercury was discovered, we may reasonably hope for as much success in the present day,—nay, more so, since it is not improbable that many of those cases which brought their victims into the grave might have been aggravated and rendered incurable by the very means employed for their relief,—a rock which our present increased knowledge would enable us to avoid.

I shall go back no farther than the year 1160, three hundred and thirty-four years before the epoch assigned for the introduction of the new disease: at this period was passed a legislative enactment in this country to regulate brothels; among other curious items of which, it is decreed—

"No stew holder is to keep any woman that has the perilous infirmity of burning:"

brenning, or burning, being the old English name for clap, employed at least as far down as the time of Henry the 8th, and probably later. In 1530, Simon Fish, speaking of priests, says,

"These be they that corrupt the whole generation of mankind in the realm, that catch the pockes of one woman and bear them to another, that be *burnt* with one woman and bear it to another, that take the leprosy from one woman to another."

Another article begins thus,

"*De his qui custodiunt mulieres habentes nephandam infirmitatem.* (Item.—That no stew holder keep noo woman wythin his hous that hath any sycknesse of brenning, but that she be putte out upon the peyne of makeit a fyne unto the Lord of a hundred shylayngs.")

A manuscript of John Arden, surgeon to Richard II. and Henry IV. defines burning to be a certain inward heat and excoriation of the urethra, and treats of phymosis and paraphymosis with their remedies; he speaks also of contumacious ulcers of the glans penis, and the great trouble of curing them. The venereal mode is designated by him *Boon-haw:* haw signifying a swelling. This disease he cured by destroying the thickened periosteum by kali purum.

Independently of these direct testimonies, and many others equally striking, there exist strong grounds for believing that a very great proportion of the cases of leprosy described by the ancients were in reality the venereal disease.

In the descriptions of some of the species are enumerated all the symptoms of the syphilis in its severest form, even ulcerations of the nose and palate; and nodes. That of the present day possesses none of these characters.

"Nobody (says Becket) ever observed *our* leprosy to be attended with falling off of the hair, hoarseness of the voice—the patient speaking as through the nose; consumption of the flesh; ulcers all over the body; corruptions of the fleshy parts and of the bones themselves; filthy ulcers of the throat, corronon, and falling of the nose: all of which are described by the ancients under the term Leprosy: besides, their disease was got by coition; but in ours, a diseased husband may cohabit with his wife with impunity."

To which we may add another very striking difference, the *libido inexplebilis,* ascribed by the ancients to their disease; whereas, in that of modern times, venereal appetite is not only wanting, but the testicles themselves wasted. The leprosy of the ancients was probably the most common and incurable disease with which they were afflicted, and it unquestionably also was frequently fatal; neither which properties are remarked in the present day, even in those places where it is more particularly prevalent. There is no disease more likely to be universal than syphilis, at a time when its treatment is not well understood. When mercury was introduced and found to be a specific for syphilis, then it was that the leprosy began to abate its terrors and became comparatively a rare disease; and that which does occasionally present itself is very different from the disease of the ancients.

Eunuchs were never, or very rarely, troubled with the leprosy. Le Prestre* tells us, that, to avoid the peril of this disease, men made eunuchs of themselves. Mezeray, the historian, gives the same testimony. The subsequent immunity from the disease could only arise from the impossibility of receiving it *in coitu.* Dr. Pitcairn remarks, that the leprosy, before the Neapolitan disease was talked of, was cured by mercury, and now, having changed its name (to syphilis), is no longer heard of.†

This opinion, that one species of the disease, generally called Leprosy, is syphilis, is not new; our countryman, John of Gaddesden, in his chapter on Leprosy, enumerates as their symptoms, ozenas, ulcers of the throat, cancerous and corrosive ulcers of the limbs: among the requisites to produce its *contagious* property, we find that the yard of the person who has the disease must be "*corrupta in substantia vel complexione vel utroque.*"

Sebastianus Aquilanus endeavoured to prove it from Galen, Avicen, Pliny, and others.

John of Gaddesden, who wrote in the 14th century, very distinctly describes the venereal disease, and even gives us a mode of preservation from the infection. "If a man (says he) wishes to preserve his yard from corruption, after connection with a woman whom he suspects of uncleanliness, let him wash himself with cold vinegar and water, or with urine, within and without the prepuce."—When treating of leprosy, he recommends a decoction of plantain and roses in wine to be made use of immediately after the venereal encounter; hence it is evident some of the leprous women have, in his opinion, the power of communicating an infectious malady to those that had carnal conversation with them. In true leprosy, we never meet with any disorder of these parts; it appears, therefore, there was a disease among them which was not the leprosy, though it went by that name. Bartholomew Granville, in 1360, describes leprosy "as comyng of

* "De Separatione ex Causa Luis Venereæ."

† "Lepra autem ante Famam Morbi Neapolitani Hydrargyro cessit, Nomenque nunc Amisit."—*Arch. Pitcairnii Dissertat. Medicæ,* 1713, *de ingressu Luis Venereæ.*

fleshly lyning by a woman after that a leprous man hathe laye by her, and also when a chylde is fedde with corrupte mylke of a leprous nouryce."

In the year 1270, wrote Gulielmus de Saliceto: "*An apostem in the groin* (says he) *is called Bubo; it sometimes arises when a sore on the yard exists from connexion with a foul woman.*" And, speaking of the affections of the penis and præputium from impure connexion, entitles his chapter, "*De Pustulis Albis vel Rubeis, & de Milio, & de Scissuris, & de Corruptionibus vel Hujusmodi quæ fiunt in Virga, vel Circa Præputium propter* Coitum cum Muliere Fœtida aut cum Meretrice, *aut ab alia Causa; Chirurg. lib.* 1. *cap.* 48.

Lanfranc, in 1290, says, "Buboes often arise in the groin on account of ulcers in the yard." The affections of the penis are called by him, Ficus, Cancer, and Ulcus: he speaks of hot and spreading pustules, occasioned by intercourse with a foul woman who had been connected with a person labouring under the disease.

Bernard Gordon, in 1300, "*De Passionibus Virgæ,*" assigns many complaints to this organ; apostems, ulcerations, cancer, &c. among the causes—"*jacere cum muliere cujus matrix est immunda.*"

Guy de Chauliac, 1360, writes, "*De Calefactione & Fœtidate in Virga propter* Decubitum cum Muliere Fœtida."

Valescus de Taranta, in 1400, says, "that ulcers and pustules of the penis are occasioned by intercourse with an impure or cancerous woman; that the pustules are received from a woman labouring under ulceration in the genitals, &c."

These quotations might be multiplied, if necessary; and, it may be proper to state, that among the causes enumerated of these affections were those of an internal kind. This will not be surprising to those who know from experience at home how much these surgeons were open to deception from their patients: it is a matter of history that the priests in these times formed a prominent part of the list of venereal patients; doubtless, when his holiness the Pope got into the fire, he would affirm that his disorder could arise from none but an inward cause, and the poor bigoted surgeon would attach implicit faith to such infallible authority.

Without any further comment, I leave my professional brethren to determine for themselves whether these are cases of genuine syphilis, or merely examples of the spurious disease.

*Camden Town; August* 1, 1818.

